# Experimental Evaluation of Unicast and Multicast CoAP Group Communication

**DOI:** 10.3390/s16071137

**Published:** 2016-07-21

**Authors:** Isam Ishaq, Jeroen Hoebeke, Ingrid Moerman, Piet Demeester

**Affiliations:** 1 Said Khoury IT Center of Excellence (SKITCE), Al-Quds University, Abu Deis, Jerusalem 51000, Palestine; 2 Ghent University—iMinds, Department of Information Technology (INTEC), Technologiepark-Zwijnaarde 15, 9052 Ghent, Belgium; jeroen.hoebeke@intec.ugent.be (J.H.); ingrid.moerman@intec.ugent.be (I.M.); piet.demeester@intec.ugent.be (P.D.)

**Keywords:** wireless sensor networks, Internet of Things, CoAP, sensors, group communication, multicast, entities

## Abstract

The Internet of Things (IoT) is expanding rapidly to new domains in which embedded devices play a key role and gradually outnumber traditionally-connected devices. These devices are often constrained in their resources and are thus unable to run standard Internet protocols. The Constrained Application Protocol (CoAP) is a new alternative standard protocol that implements the same principals as the Hypertext Transfer Protocol (HTTP), but is tailored towards constrained devices. In many IoT application domains, devices need to be addressed in groups in addition to being addressable individually. Two main approaches are currently being proposed in the IoT community for CoAP-based group communication. The main difference between the two approaches lies in the underlying communication type: multicast versus unicast. In this article, we experimentally evaluate those two approaches using two wireless sensor testbeds and under different test conditions. We highlight the pros and cons of each of them and propose combining these approaches in a hybrid solution to better suit certain use case requirements. Additionally, we provide a solution for multicast-based group membership management using CoAP.

## 1. Introduction

The number of devices connected to the Internet is rapidly increasing. On the one hand, within traditionally-connected domains, the number of devices and the variety of device types are gradually increasing. On the other hand, traditionally unconnected domains are discovering the benefits of the Internet and are joining it continuously. The resulting Internet is now referred to as the Internet of Things (IoT) to stress the fact that it is connecting all sorts of things, not just people, computers and smart phones.

It is expected that the IoT will soon contain more embedded devices than the traditionally-connected devices. These embedded devices are often constrained in their resources since they are optimized for low cost and/or battery operation. Most commonly, these devices have constraints in terms of the available amount of RAM, ROM and CPU power. They are often required to consume very little power, since they are often battery powered or rely on energy harvesting. Ideally, these devices should run for years without the need for costly battery replacements. These device constraints impose constraints on the networks in which these devices operate. These networks typically have lower bandwidths and higher error rates than common Internet networks. These networks are usually referred to as Low-power and Lossy Networks (LLNs).

The early integration of constrained devices into the Internet resulted in a situation that was similar to what computer networks looked like about two decades ago: islands of computers communicating with their own protocol interconnected by complex multi-protocol gateways, which are inherently complex to design, manage and deploy. The resulting end-to-end Internet Protocol (IP) architecture has tackled those issues and has been widely accepted as the only alternative to design scalable and efficient networks of large numbers of communicating devices. For the future IoT, scalability, stability and efficiency are even more important than ever. IP therefore is the future proof choice for the IoT [1]. On top of that, past efforts have shown the feasibility of implementing a minimal footprint IPv6 stack on constrained devices (100 kB ROM, 10 kB RAM).

A logical consequence of this evolution was the investigation of also adopting web service technologies on top of such end-to-end IP connectivity to avoid isolated islands in terms of APIs and semantics. Web services are at the basis of the success of today’s Internet. They are ideal for building distributed applications through communication using well-defined message sequences and data formats in a system-independent way. Web-style applications and IoT applications share many of these basic communication properties; they are composed of separate systems that exchange data. Different forms of web paradigms exist, but research has shown that the REST paradigm is most suitable for constrained devices [2].

As a result of device and LLN constraints, the use of standard Internet protocols becomes often unfeasible, since these protocols were not designed to accommodate those constraints. In the past few years, there were many efforts to enable the extension of the Internet technologies to constrained devices. Most of these efforts were focusing on the networking layer, e.g., IPv6 over Low power Wireless Personal Area Networks (6LoWPAN) [3] and IPv6 Routing Protocol for Low-Power and Lossy Networks (RPL) [4]. More recently, work has started to allow the integration of constrained devices in the Internet at the service level. The Internet Engineering Task Force (IETF) Constrained RESTful Environments (CoRE) working group [5] has realized the Representational State Transfer (REST) architecture in a suitable form for the most constrained nodes and networks. As a result, the Constrained Application Protocol (CoAP) [6] was introduced, a specialized RESTful web transfer protocol for use with constrained networks and nodes. CoAP realizes a subset of REST that is common with the Hypertext Transfer Protocol (HTTP), but is optimized for Machine-to-Machine (M2M) applications, such as smart energy and building automation. With the introduction of CoAP, a complete open standard networking stack suitable for constrained devices is now available [7]. Constrained devices are turned into embedded web servers that make their resources accessible via the CoAP protocol.

In many IoT application domains, the devices need to be addressed in groups in addition to being addressable individually. For example, in a smart building, it should be possible to manually or automatically control the opening of all window shutters at a certain side of the building based on the position of the Sun. This operation of the shutters as a group should not hinder the ability of the user to control a single shutter individually or to control only those shutters that belong to his or her room. Two main approaches are currently being proposed in the IoT community for CoAP-based group communication. The main difference between these two approaches lies in the underlying communication type: multicast versus unicast. The experimental standard for CoAP group communication [8] relies on Internet Protocol Version 6 (IPv6) multicasts, while our approach proposed in [9] relies on IPv6 unicast messages.

The main contributions of this article are the following:

- An experimental evaluation of CoAP-based multicast and unicast group communication solutions using two wireless sensor testbeds representing different testing environments. The first testbed is located in a large, shielded room, which allows testing under controlled environments. The second testbed is located inside an operational office building and thus allows testing under normal operation conditions. We highlight the pros and cons of each of the group communication approaches. To our knowledge, no other work has done such evaluations at a large scale.
- A novel hybrid group communication approach providing flexibility regarding which method, unicast or multicast, is being used. This flexibility can better deal with varying use case requirements.
- An extension of our group management framework enabling the use of CoAP for the management of the multicast group membership. Using this simple extension, it becomes possible to ask CoAP-enabled devices to join and leave multicast groups without the need for manual intervention or a dedicated management protocol. This is an essential feature in dynamic contexts, where group membership is changing frequently.

The remainder of this article is organized as follows. In Section 2, we provide the motivation for this work and briefly show the need for group communication in various application domains of the IoT We provide a brief introduction to CoAP and introduce the different approaches for CoAP group communication. We then discuss related work in Section 3. In Section 4, we introduce our CoAP group communication implementation and evaluate it in Section 5 before concluding this paper in Section 6.

## 2. Group Communication

In this section, we provide the motivation for this work. We start by briefly showing the importance of group communication in various IoT application domains, followed by an introduction to CoAP-based group communication. Finally, we introduce our hybrid group communication, which is the new contribution of this paper.

### 2.1. Importance of Group Communication in the IoT

The basic CoAP interaction model is based on one-to-one communication, i.e., a client is exchanging messages with a server. The basic interaction model covers a wide range of interaction patterns that are found in various fields of the IoT. However, in many use cases, a one-to-many communication pattern is convenient or even essential for various reasons. Sometimes, the data collected from sensors might not be sufficient to get the complete information about the monitored object. In other cases, it is desired to get the information from more than one source to increase accuracy or reliability. Sometimes, data from many sources need to be collected and processed before they can be used. Likewise, it is often needed to control more than one actuator at once to make the complete object act as desired. This need to communicate with groups of objects is obvious in many IoT scenarios and is very well recognized in the IETF, as it is part of the charter of the IETF CoRE Working Group [10].

The relevance of multicast communication for wireless networks for Building Automation Systems (BASs) has been identified in [11]. In this paper, we focus on group communication in BASs, which is in line with the experiments we conduct on the testbeds in Section 5.

There are many ways to group resources based on the application requirements. Table 1 shows some possible types or motivations for resource grouping in BASs.

Within this context, we have defined the requirements and goals for CoAP group communication, which are described in detail in [9]. We have motivated their importance in the context of IoT applications, constrained devices and LLNs. For the completeness of this article, we summarize the requirements for group communication in Table 2.

### 2.2. CoAP-Based Group Communication

There are two main approaches to allow CoAP to be used for the interaction with groups. The first approach relies on IPv6 multicasts and was adapted by the IETF CoRE working group. We have proposed an alternative approach in [9] that relies on IPv6 unicasts. These two communication methods have different characteristics that heavily influence the respective approaches that use them. In this subsection, we discuss the different approaches to realize CoAP-based group communication, after briefly introducing the key features of the CoAP protocol.

#### 2.2.1. The Constrained Application Protocol

Recent research on embedded web services is laying the ground for a better integration of sensor resources into the service web. Since the dominating web protocol HTTP is too complex, the IETF Constrained RESTful Environments (CoRE) working group has designed a simpler web protocol, CoAP, and defined it in Request For Comment (RFC) 7252 [6]. CoAP uses the same RESTful principles as HTTP, but it is much lighter, so that it can run on constrained devices [2,12]. To achieve this, CoAP has a much lower header overhead and parsing complexity than HTTP. It uses a four-byte base binary header that may be followed by compact binary options and payload.

The CoAP interaction model is similar to the client/server model of HTTP. A client can send a CoAP request, requesting an action (specified by a method code) on a resource (identified by a Uniform Resource Identifier (URI)) on a server. The CoAP server processes the request and sends back a response containing a response code and payload.

**Figure 1:**
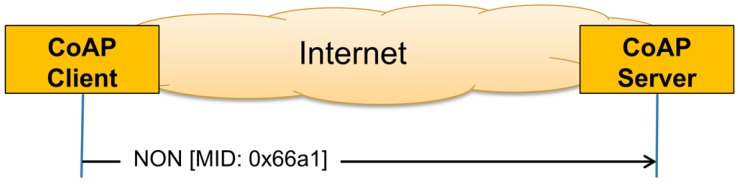
Example of CoAP Non-confirmable Message (NON) exchange. A Message ID (MID) is needed in the header for duplicate detection.

A message that does not require reliable transmission can be sent as a Non-confirmable Message (NON). This type of messages is not acknowledged, but has a message ID for duplicate detection (see Figure 1). Unlike HTTP, CoAP deals with these interchanges asynchronously over a datagram-oriented transport, such as User Datagram Protocol (UDP), and thus, a NON might get lost without the client and the server noticing it. Multicast CoAP requests are sent using NONs.

**Figure 2:**
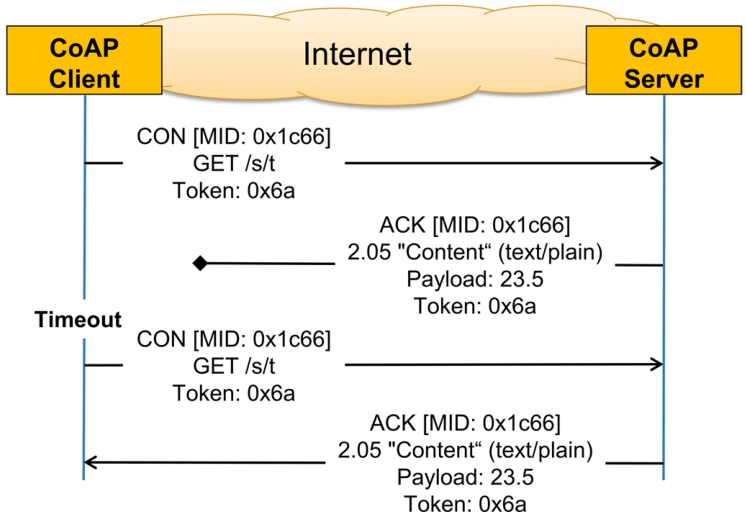
Example of CoAP Confirmable Message (CON) exchange. If the client does not receive an ACK for its CON within a certain time, it retransmits the same CON again until it gets acknowledged or until the client runs out of retransmission attempts.

Since UDP does not provide reliable communication, optional reliability is supported within CoAP itself. This is done by using Confirmable Messages (CONs) to implement simple stop-and-wait retransmissions with exponential back-off. Figure 2 shows an example for a confirmable message exchange. The Message ID (MID) must be the same in order to match Acknowledgment Messages (ACKs) with CONs and for duplicate detection. The token must be the same in order to match replies with requests. If the client does not receive an ACK for its CON within an initial back-off time, it retransmits the same CON again. By default, the initial back-off is set to a random time between 2 s and 3 s. This means that if a reply to a confirmable packet is not received within the initial back-off time, the CoAP sender will double the initial back-off time and retransmit the packet. If a reply to the first retransmission is not received, CoAP will again double the back-off time and retry the transmission until MAX_RETRANSMIT (by default, four) is reached. If no reply is received after expiry of the back-off time of the last retransmission, the client will be notified about the error condition.

Finally, CoAP supports optional security by using Datagram Transport Layer Security (DTLS) [13].

#### 2.2.2. Multicast Group Communication

The IETF CoRE working group has recognized the need to support a non-reliable multicast message to be sent to a group of devices to manipulate a resource on all of the devices in the group. Therefore, they have developed the “Group Communication for CoAP-RFC 7390” [8], which provides guidance for how the CoAP protocol should be used in a group communication context. Group communication refers to sending a single CoAP non-confirmable message to all members of a specific group by utilizing UDP/IP multicast for the requests, and unicast UDP/IP for the responses (if any). This implies that all of the group members (the destination nodes) receive the exact same message.

The use of multicast is efficient in sending the requests, but does not affect the number of responses sent by the members, since these are sent as unicasts. However, the use of multicasts has its limitations and challenges:

- The most prominent limitation is the lack of reliability, which makes it not suitable for all use cases.
- Another important limitation is the cache-unfriendly nature of multicasts, preventing the possible reduction of requests and replies by utilizing caches. Depending on the use case and network topology, the reduction of packets as a result of using a cache can be better than the reduction obtained from using multicasts.
- Furthermore, multicasts are not useful when a single user action needs to trigger different sensor requests, since one multicast request delivers the same message to all group members.
- Secure communication with the group members is not possible, since all communication based on this RFC operates in CoAP NoSec (No Security) mode.
- Finally, the use of multicast requires multicast support in the network. Typically, IP multicast relies on topology maintenance mechanisms to discover and maintain routes to all subscribers of a multicast group. However, maintaining such topologies in LLNs may not be feasible given the available resources. As a result, special multicast protocols have been proposed for the use inside LLNs. For example, the “Multicast Protocol for Low power and Lossy Networks (MPL)” Internet draft uses the Trickle Multicast (TM) algorithm to manage message transmissions for both control and data-plane messages and avoids the need to construct or maintain any multicast forwarding topology [14]. An alternative is the Stateless Multicast RPL Forwarding (SMRF) algorithm, which according to [15] achieves significant delay and energy efficiency improvements at the cost of a small increase in packet loss. Regardless of the used multicast protocol, all nodes on the path between the senders and receivers must be extended to support the protocol.

#### 2.2.3. Unicast Group Communication

In our previous work [9], we have introduced our alternative unicast-based group communication solution for CoAP-enabled devices. Our aim was to create an intermediate level of aggregation to be able to easily manipulate a group of resources across multiple smart objects. Such a group of resources is called an entity, and the resources themselves are called the entity members. An entity can be created, used or manipulated through a single CoAP request.

**Figure 3:**
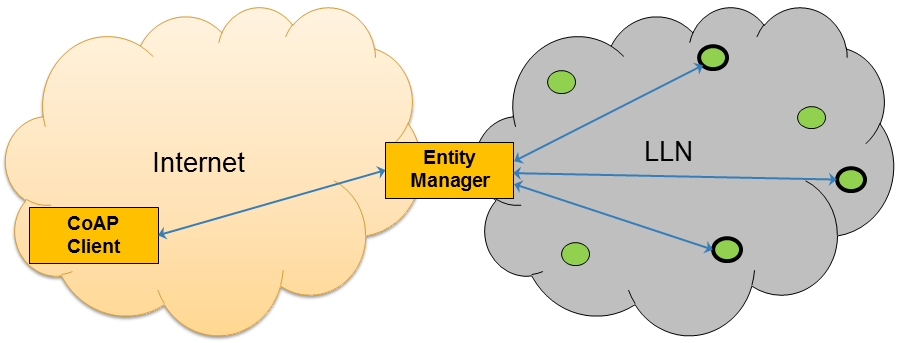
Clients create entities consisting of several smart object resources on the entity manager.

We call the component that manages the entities the Entity Manager (EM). This component, which can reside, e.g., on the border Gateway (GW) of the LLN, is responsible for maintaining entities that are created from groups of resources residing on CoAP servers (i.e., sensors and actuators) inside the LLN. Clients on the Internet can interact with an EM to create new entities and/or customize how these entities should behave. The EM exposes entities to the Internet as any other regular CoAP resource. Figure 3 shows an overview of the involved components. The EM acts as a “proxy” between the client and the constrained devices. Client requests are sent to the EM, which analyzes and verifies the requests and then issues the appropriate requests to the constrained devices using CoAP. Once the EM receives responses from the constrained devices, it combines them according to the needs of the client and sends back an aggregated response to the client.

Since an entity is exposed by the EM as yet another CoAP resource, entities can be members in other entities. This makes it possible to construct nested entities that can, for example, reflect a certain hierarchy of the members.

When a client tries to create a new entity consisting of a group of resources inside LLNs, the EM performs a sanity check on the request in order to make sure that the resulting entity would make sense. We call this sanity check the entity validation.

Furthermore, we have introduced in [9] the notion of profiles for the created entities. The use of entity profiles allows the client to specify in more detail how the entity should behave (e.g., if it should use confirmable or non-confirmable CoAP messages) and, through updating the profile, allows manipulation of this behavior. By building upon standardized concepts, the impact on the constrained devices was limited.

An important advantage of our approach is its reliability, since it uses unicast messages that can make use of the CoAP built-in reliability mechanism. Its main disadvantage is the increased communication overhead to achieve this reliability. For more details, we refer the reader to [9].

### 2.3. Hybrid Group Communication

In the previous subsection, we have briefly described the two main approaches for CoAP group communication. The first approach uses CoAP NON over IPv6 multicast, and the second one uses CoAP CON over IPv6 unicasts. We have shown that each approach has its strong and weak points. The multicast approach is generally fast and has little overhead, but is not flexible and not reliable and, thus, cannot be used when reliability is required. One the other hand, the unicast approach is reliable and flexible, but is often slower and has more overhead.

In this paper, we extend our previous unicast-based solution with multicast support. We do so by maintaining the overall architecture of our approach. Clients still create entities on the Entity Manager (EM) to represent the groups and have to query the CoAP group resource on the EM to access the group. However, at creation time, clients can specify the communication type between the EM and the entity members (i.e., multicast or unicast). We have extended our existing solution so that all of its features become valid for entities communicating by using multicast as they are valid for entities using unicasts. In this subsection, we discuss the most important features and how they are extended to allow entities to use multicast communication.

#### 2.3.1. Entity Profile

Resource profiles can be used to express the capabilities of a CoAP server and its resources [16]. In [9], we have extended the resource profiles concept with entity-specific fields. By using entity profiles, it becomes possible to customize the default behavior of the entity. These entity profile fields can be provided by the client upon entity creation time. If no values are provided, the EM will use default values for the newly-created entity. Clients can either use this default behavior or change any of those fields each time they use the entity by adding URI queries to the request. Following is a list of the entity profile fields that are relevant to this current work.

- Entity resources (er): a list of the individual resources out of which the entity is composed.
- Entity message type (emt): specifies the message type to be used for communication between the EM and members. Possible values are con(confirmable) and non (non-confirmable). The default is con.
- Entity message type (ect): specifies the communication type to be used for communication between the EM and members. Possible values are unicast and multicast. The default is unicast. Whenever ect is set to multicast, the EM sets emt to non, since CoAP supports only non-confirmable multicast requests. Please note that this is the only profile field that can be set only at entity creation time and cannot be set at usage time by using URI queries. The reason for this is that changing the communication type implies changing the multicast group membership (see Section 2.3.2), which we consider as a configuration task that should not be done on the fly.
- Entity number of replies (enr): specifies the number of replies that should be received from the members (member replies) before sending a reply to the client (client reply). This makes it possible not to wait for all members to reply. The default behavior is to wait until all replies have been received or have timed out. For example, the entity could consist of five members, but a reply from any three of them can be considered sufficient for the client, and thus, the EM does not need to wait until it receives replies from all group members. This does not just increase the speed of the client reply, but also makes the group tolerant to failures. This feature is of particular importance, since the client could set enrto one. In this case, the multicast entity is very similar to anycast communication where only one node of a potential group of receivers will respond. For anycast communication, the topologically-nearest node will reply. For group communication with enr equal to one, the EM will send a multicast request or several unicast requests to the group members and will send a single reply to the client as soon as the first reply from any of the group member arrives. This is an important feature, since RPL does not support anycast routing, and thus, this communication pattern was not yet available for the constrained nodes.
- Entity operation (eo): The operations that can be performed on the results obtained from the members. The operation is used to combine replies received from all the members (or the number of replies specified by enr) into one reply to the client. If at the time of querying the entity, the client does not specify which operation to use, the first operation listed in this field will be used. Currently, the following entity operations are supported:
  – List (lst): A list of replies received from the members, without any arithmetic processing. This is the default behavior if no entity operation was specified.
  – Average (avg): The average value.
  – Minimum (min): The minimum value.
  – Maximum (max): The maximum value.
- Delay between requests (delay): specifies the delay that should be injected between the requests sent from the EM to the members. The default is zero, and thus, the EM will send the requests as fast as it can.

#### 2.3.2. Group Membership Management

Another important feature that our solution provides is the multicast group membership management. This means that whenever a multicast entity is created, the EM assigns an IPv6 multicast address to the entity and automatically requests the individual members to join the multicast group. The EM verifies that the members have joined the group and informs the client about the assigned multicast address. Since the assigned multicast addresses can be globally routable, the client can communicate with the multicast group directly without contacting the EM. In order for members to be able to join the group, the members’ constrained CoAP implementation has been extended with a new resource for group management. This approach essentially implements group communication as proposed by IETF CoRE in the groupcomm draft, but with the addition of group management functionality through the entity manager. Once the group configuration has taken place, any client can interact with the group using its multicast address without the need to be aware of the presence of the EM. In addition, once all management has taken place, the EM can disappear from the network, leaving behind an operational network with configured multicast group communication possibilities. A mixed form is also possible, where clients use unicast to go to the EM, but the EM uses multicast to go the members. This way, a client can still benefit from other EM features, such as response processing. Details about the group membership management implementation will be provided in Section 4.1.

#### 2.3.3. Summary

In summary, our proposed hybrid group communication solution for CoAP is an extension to our previously proposed unicast-based solution. By extending it with multicast support, we achieved the increased flexibility of our solution. It is now up to the creator of the entity to decide whether communication reliability with the members is essential. When it is not essential and the user selects to create a multicast entity, the EM automatically configures the multicast group and lets the members join it. This minimizes manual and error-prone group membership configurations. Since group membership management is done via unicast CoAP messages, it is reliable, and the user can be informed about its results.

## 3. Related Work

As mentioned in the Introduction, the basis of our work is the newly-standardized Constrained Application Protocol (CoAP) [6] and the two main approaches to realize CoAP group communication.

The first approach was published as an experimental standard for CoAP group communication [8] by the IETF CoRE working group, who was behind the standardization of CoAP itself. This standard specifies how CoAP should be used in a group communication context. It provides an approach for using CoAP on top of non-reliable IP multicast. The use of multicast is useful for many IoT scenarios, such as for service discovery. The authors of [17] proposed an alternative lightweight forwarding algorithm for efficient multicast support in LLNs targeting service discovery for duty-cycled constrained devices. Certainly, the use of multicasts allows reducing the amount of requests in the LLN, by sending one request to several destinations at the same time. However, the use of multicast has limitations, such as not being cache-friendly and not supporting secure communication (see Section 2.2.2).

An alternative multicast-based group communication solution was presented in [18]. This work presented a concept of a web service-based communication stack that uses transient link-local IPv6 multicast addresses for process data exchange between nodes by binding a shared network variable to the IPv6 multicast address. By doing so, the authors were able to reduce the CoAP multicast size by eliminating the need to include the URI path to identify the group communication resource on the members. URI identifiers are text-based. As such, they can be verbose and have a severe impact on the CoAP header size, since no compression can be provided for them. Certainly, this approach has several advantages, such as eliminating the need for a control unit, offering a lower power consumption than using unicasts and its suitability in many non-critical use cases (due to the lack of reliability of multicasts). However, since this approach is based on IP multicast, it exhibits the limitations of multicasts as discussed in Section 2.2.2. In addition, to our knowledge, apart from simple performance evaluations, no larger scale evaluations of the performance of multicast solutions have been published. Consequently, at of the time of writing, clear insights into the behavior and performance of multicast operations in LLNs are missing.

The second approach is to rely on unicast messages to realize CoAP group communication. In [9], we have introduced a unicast-based group communication solution that uses the notation of entities to represent groups of CoAP resources. We have evaluated it using simulations and small-scale demonstrations and showed that it complements multicast-based solutions when the reliability of the communication is desired. Another unicast-based group communication solution is called SeaHttp and was presented in [19]. The authors propose to extend CoAP with two additional methods (BRANCH and COMBINE) to allow members to join and leave groups without the need for a separate group manager. This means that members should have the intelligence to know which group they should join/leave. Constrained devices will not have this intelligence, so again, a “manager” will be needed to inform the devices so they can take appropriate actions. Furthermore, BRANCH and COMBINE may be able to reduce the number of messages; however, the trade-off is the need to implement a new mechanism. It is better to use an approach that can be plugged into any existing network without major modifications (or at least not a modification to every node). Finally, this approach does not have the flexibility we target, since group members have to be reprogrammed with the groups they should join each time the requirements of the user changes.

To our knowledge, these are the only works that explore communication solutions for interacting with a group of CoAP-enabled constrained devices. Next to these, there exist other solutions to realize group-like communication in constrained environments without using CoAP. For example, the Message Queue Telemetry Transport (MQTT) protocol is another application layer protocol designed for constrained devices [20]. MQTT uses a topic-based publish-subscribe architecture, i.e., clients utilize the services of a broker to subscribe to topics and get all of the messages that are published to that topic. Unlike CoAP, MQTT relies on TCP as the underlying transport protocol and, thus, inherits its reliability. While the base CoAP does not provide any Quality of Service (QoS), MQTT provides its own QoS mechanism. MQTT provides its own way of group communication, by allowing multiple publishers to publish to the same topic and by allowing multiple clients to subscribe to it. This can be seen as a form of group communication, which exhibits some similarities with our proposed approach. However, it does not adhere to the REST principles that are commonly used for the Internet, and additionally, it does not provide the possibility to aggregate and manipulate notifications that are sent to the clients.

MQTT for Sensor Networks (MQTT-SN) is an adapted version of MQTT that is aimed at embedded devices and Wireless Sensor Networks (WSNs) [21]. MQTT-SN extends MQTT beyond the reach of TCP/IP networks by taking into consideration the peculiarities of wireless communication environments. Similar to our solution, MQTT-SN relies on the presence of a gateway to act as a proxy between the clients and the servers (brokers in the MQTT case). However, like MQTT, MQTT-SN does not adhere to the REST principles.

## 4. Implementation

The key in our group communication solution is the EM. We have implemented the EM functionality using the CoAP++ framework [22] and typically run it on the GW of the LLN. The CoAP++ framework and the EM implementation on top of it have been realized in Click Router, a C++-based modular framework that can be used to realize any network packet processing functionality [23].

As group members, we have used Zolertia Z1 [24] and Rmoni RM090 [25] boards. On these boards, we have run a development version, a pre-release of Contiki 3.0; snapshot from the master branch on GitHub on 2 July 2014 of the Contiki 3.0 operating system [26]. This version of Contiki was the most recent development version when we started our experiments. It supports multicast and includes a stable implementation of CoAP, namely the Erbium CoAP server [27].

### 4.1. Multicast Group Management Using CoAP

By default, Contiki nodes join four multicast groups when multicast is enabled on them. All of these groups have link-local scope, and thus, the IPv6 multicast addresses of all of them start with ff02: (Table 3). The first three addresses are the same for all nodes, since these are generic addresses representing all nodes, all routers and all RPL-enabled nodes on the local network segment. The last address is unique for each node and depends on the node ID. This address is called the solicited-node multicast address and is created by taking the last 24 bits of the nodes unicast address and appending this to the prefix ff02::1:ff00:0/104 [28]. The solicited-node multicast address is used in neighbor discovery for obtaining the Layer 2 link-layer addresses of other nodes [29].

In addition to the four default multicast groups that the nodes join automatically, the Contiki multicast implementation allows specifying other multicast groups to join as needed. However, the maximum number of groups to join is set at compile time. By default, a node can join only one group in addition to the groups listed in Table 3. This maximum number of groups that a node can join can be changed at compile time. For each additional multicast address, the node requires 18 bytes of RAM. When using the nodes in a static setting, the multicast address(es) can be hard coded when the node is programmed. However, since many group communication scenarios require frequent group membership changes, it is essential to have a solution that allows changing a node’s group membership on the fly without the need to reprogram it. We have provided such a solution using the same technology that we use to communicate with nodes, i.e., using CoAP. By using CoAP for this task, as well, we eliminate the need for a dedicated group management protocol and can keep the solution lightweight.

In order to offer a RESTful interface for multicast group membership on the constrained devices, we extended the Erbium implementation on these devices with a new CoAP resource: /mc. This resource can be used to list the multicast addresses of the groups that a node has joined, to join a new group and to leave a group. Table 4 documents the interface to the group membership management resource.

The footprint of the multicast group membership resource is small. It requires only 1408 bytes of ROM and 28 bytes of RAM when compiled with gcc 4.6 for the RM090 nodes. In Table 5, we provide resource availability for the nodes that we used in our tests along with the resource requirements of the two available multicast implementations and our multicast membership management resource.

### 4.2. EM Multicast Extensions

We have extended our unicast-based solution to support communicating with the group members using IPv6 multicasts. We have also extended the existing features of our solution to support this new communication type. More specifically, we have defined a new profile field ect to specify the entity communication type. This field can take two possible values, namely ect = ‘‘unicast’’ or ect = ‘‘multicast’’.

When a multicast entity is created, the EM assigns a new multicast group address to the entity and adds another field to the entity profile with the name mcastURI. This field specifies the URI to which a multicast address can be sent, when it is desired to communicate to the multicast group without going through the entity resource on the EM. This URI contains the IPv6 multicast address of the group and the resource name on the individual members, e.g., coap://[ff1e::89:1]/s/t. Since the resource name has to be the same across all group members, the EM adds this check to the validation process of multicast entities. In our implementation, we used the following IPv6 multicast addresses ff1e::89:xx where the first 16 bits of the address (ff1e) indicate that the address is a non-permanently-assigned (“transient” or “dynamically” assigned) multicast address with global scope, and the xx is a 16-bit sequential number.

The EM then uses the group membership management resource on the nodes to let them join the newly-assigned multicast address. The communication with the entity management resource on the constrained devices is done via unicast CON messages to ensure reliability. The group creation is considered successful only if all required members successfully join the group. In any case, the group creator is informed about the results of the multicast group join requests. Similarly, when a multicast group is deleted, all members are sent a unicast request to leave the multicast group, and the deleter of the group is informed about the results.

**Figure 4:**
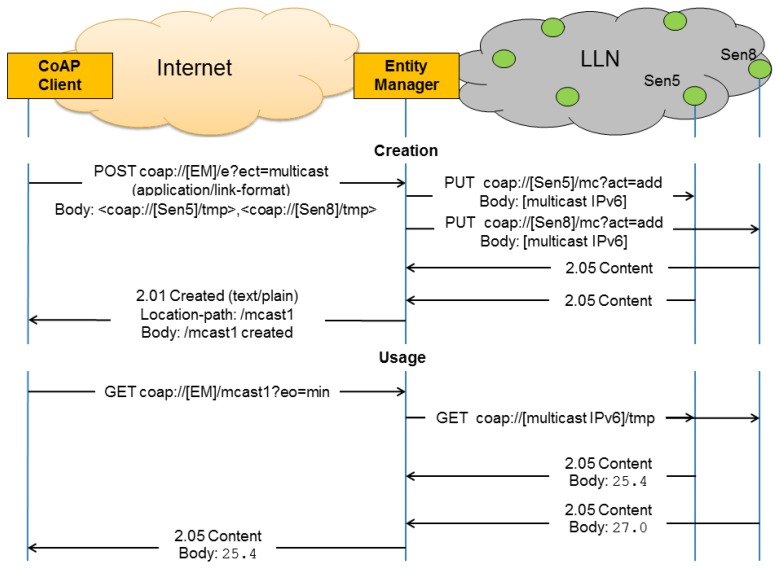
An example of the creation and usage of a multicast entity. LLN, Low-power and Lossy Network.

An example of the creation and usage of a multicast entity is shown in Figure 4, where the client requested the creation of a multicast entity with two members. Consequently, the EM assigned a multicast address to the group and sent two unicast messages to the members asking them to join that multicast address. Once the EM received replies from all members that they joined the multicast group successfully, the EM notified the client about the address of the multicast group. Later, when the client queried the EM about the multicast group, the EM sent a multicast request to the group’s multicast address, received two replies and sent back a combined reply back to the client.

## 5. Evaluation

When we evaluated our approach in [9], we have used our Demo box for the demonstration of the functionality [30] and the Cooja network simulator, which is part of the Contiki operating system, for the performance evaluations. The simulation environment enabled the initial evaluation of the performance of our solution for varying entity sizes and number of hops to the entity resources. However, evaluation on larger real-life testbeds is required for validating the simulation experiments and for conducting experiments in denser and more realistic (e.g., Wi-Fi interference) environments. In this section, we will present an extensive evaluation of both multicast- and unicast-based solutions on two wireless sensor testbeds with different characteristics (Section 5.2 and Section 5.3). Before doing so, we first start by evaluating the functionality of the new extensions to our solution in (Section 5.1).

### 5.1. Functional Evaluation

The two main features that we have added to our approach as originally presented in [9] are the support for multicast entities and the provisioning of more advanced features, such as the nesting of entities. In this subsection, we functionally evaluate these two extensions.

#### 5.1.1. Multicast Entities

**Figure 5:**
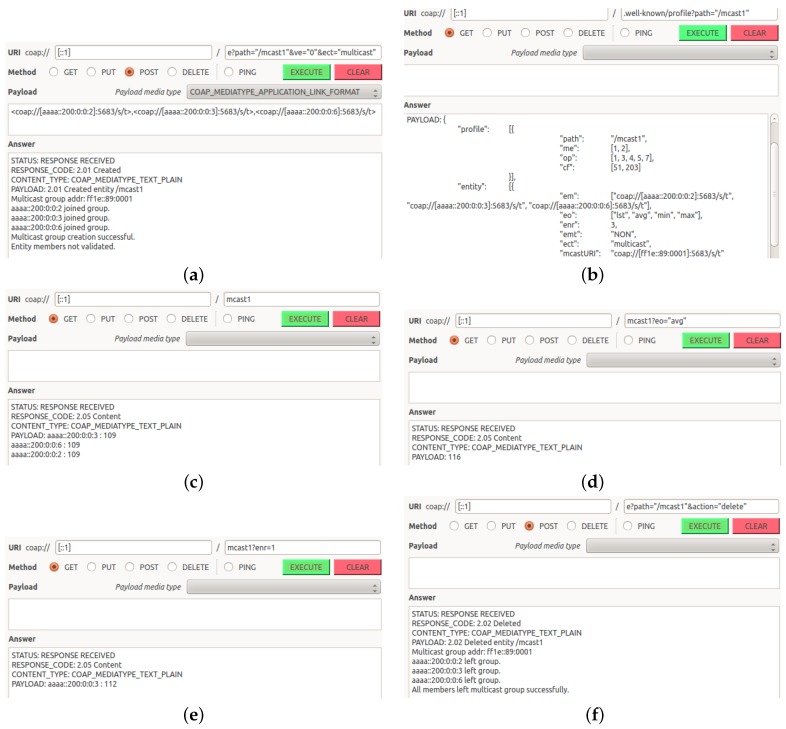
Screenshots using the CoAP++ client GUI to create, query and delete a multicast entity of three members. (**a**) Creating a multicast entity mcast1; (**b**) profile of mcast1; (**c**) querying mcast1 with default properties; (**d**) querying mcast1 with entity operation avg; (**e**) using mcast1 as an anycast entity; (**f**) deleting the entity mcast1.

The functionality for creating, validating, using and deleting multicast entities has been implemented as described above. In this subsection, we demonstrate the main functionalities of the implementation using a series of screenshots covering the life cycle of a multicast entity (Figure 5). These screenshots are taken using the CoAP++ client Graphical User Interface (GUI).

In Figure 5a, the client initiates a request to create a multicast entity consisting of three members. The members are three temperature resources on RM090 sensors. The EM first assigns a unique IPv6 multicast address (ff1e::89:1) to the group and then asks all members to join this multicast group. The entity is created successfully and is ready for use since all members have reported that they have successfully joined the multicast group.

Next, the client checks the profile of the newly-created entity (Figure 5b). The profile confirms that the entity uses multicast for communication (ect: multicast). Consequently, CoAP NON messages will be used to communicate with the members (emt: NON). The profile also shows that the entity supports four entity operations: lst, avg, min and max, where lst is the default operation, as it is the first operation in the list of supported operations. It further shows that the EM will wait until it receives three replies from the members before sending the combined reply to the client (enr: 3).

Next, the client queries the newly-created entity without specifying any URI queries, and thus, the default entity properties (entity operation, entity number of replies, etc.) are applied. Figure 5c shows how the client receives a reply containing all members’ values.

Now, assume the client is not interested in the individual values of all members. As part of the request, the client uses the entity operation avg as part of the URI query to obtain the average temperature of all three sensors. By doing so, the EM now calculates the average and only sends the calculated value to the client (Figure 5d). The disadvantages of this approach is that the EM will wait until it receives replies from all members before sending the reply to the client and that it might not get all replies since multicast communication is not reliable. Thus, the client decides to set the entity number of replies to a single reply (enr = 1). By doing so, the EM will send a reply to the client as soon as it gets a reply from any of the members (Figure 5e). This way, the EM emulates anycast behavior, which is not supported by RPL.

To complete the life cycle of this multicast entity, we show how the client deletes it in Figure 5f. As a result of deleting the entity, the EM requests that all members leave the multicast group, waits until the members confirm that they have left the group and conveys the results to the client. To avoid further confusion (e.g., bookmarked URIs containing the multicast addresses), the EM will not use the same multicast address for new groups unless it runs out of addresses.

#### 5.1.2. Extended Entity Features

Our solution is flexible and can be used to build more complex entities than we have shown so far. Since entities have URIs and behave in the same way as any other CoAP resource, entities can themselves become part of other entities to build a hierarchy. To illustrate this, consider the following scenario.

A large cold storage building consists of several floors with several cold storage rooms in each of them. In each room, there are at least three battery-operated CoAP-enabled wireless temperature sensors. For quality assurance purposes, it is required to centrally log the average temperature of each room every hour.

The most straightforward solution is to query all of these sensor individually and to add all of the intelligence about the distribution and the processing of the individual values in the client application. However, using our solution, this can be accomplished in a hierarchical way as follows:

1. Per room, create a room temperature entity containing all temperature sensors. Since every room has at least three sensors and is required to get the average temperature, the entity should use the entity operation eo=avg. Since the sensors are battery operated, they are likely to have a Radio Duty Cycles (RDC) with long sleep periods. Further, as only the average is needed, the administrator can decide for each room how many values are needed to build the average and set the entity number of replies accordingly, e.g., enr=2. To reduce the communication overhead, multicast communication with the sensors can be used, i.e., ect=multicast.
2. Per floor, create a floor temperature entity containing all of the room entities of the floor, i.e., as members the URIs of the previously created room temperature entities are used. Since it is expected to have values for all rooms, this entity can be created with default behavior, which implies eo=lst, enr="number of entity members" and ect=unicast.
3. Create one building temperature entity containing all floor temperature entities in a similar way as the floor temperature entities.
4. Query the building temperature entity to get results for all of the rooms in the building. The client can decide in which media type to get the reply. Using plain text might be useful for the client to view or to include in reports. Since in this scenario the data should be logged, it is more likely to request the data in a more structured format, such as SENML5+JSON [31], so it becomes easier to extract and add the values to a database.

The example above can be easily extended in the hierarchy (e.g., building, campus, etc.). Entities created for one reason can be reused for other purposes, e.g., the room temperature entities can be used by the respective cooling system to control the temperature in rooms. This is true even if the entity should behave in a different way than it was created. For example, to troubleshoot heat distribution inside a room, a technician can query the same room temperature entity, but requesting to get a list of all individual sensor values in order to build a room heat map. From this example, one can see that entities can be configured and combined in flexible ways to suit the clients’ needs.

### 5.2. Performance Evaluation on a Wireless Sensor Testbed

**Figure 6:**
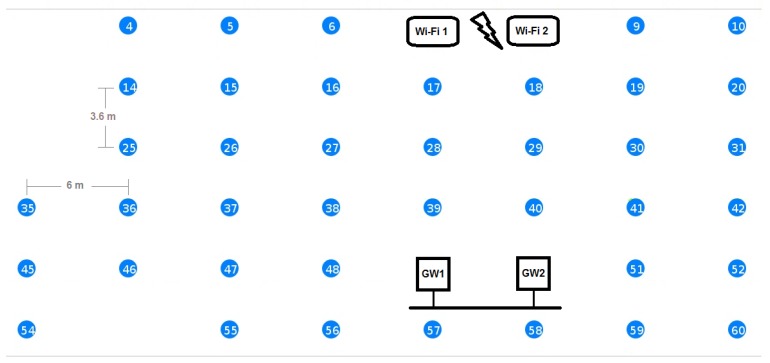
Experimental setup at w-iLab.t Zwijnaarde: generic wireless testbed. The circles represent the location of the nodes.

We have conducted the majority of the evaluation tests on the w-iLab.t wireless sensor testbed in Zwijnaarde [32]. This testbed provides a controlled test environment in a large (66 m × 20 m) open room with 60 fixed nodes and 15 mobile nodes. Each node includes sensors (Rmoni RM090 and Zolertia Z1) and Wi-Fi (IEEE 802.11a/b/g/n). In our experiments, we have used up to 40 fixed nodes as sensor nodes, two fixed nodes as a sensor network GWs and two other fixed nodes to generate interfering Wi-Fi traffic when needed. Figure 6 shows the part of the testbed that we used during our experiments. The other nodes were idle during the experiments to make sure that they do not cause extra interference and are not shown in Figure 6 for clarity. The two GWs are connected via an Ethernet link. When both GWs were used, they operated on different IEEE 802.15.4 Radio Frequency (RF) channels. However, in most experiments, we used only GW1 and kept GW2 idle in order to not interfere with the running experiments. In all of the performance evaluation experiments, we used the Rmoni RM090 boards with Contiki. We have used RPL as the routing protocol and enabled the SMRF multicast engine. We did not use any RDC. The GWs are running the example rpl-border-router provided by Contiki, and therefore, they are the RPL DODAGroots for their subnets, delegate the global IPv6 prefix and route traffic to and from the constrained networks. All other nodes run the Erbium server and also have RPL enabled. In Table 6, we summarize the most important settings for Contiki and the used protocols.

In the following subsections, we present the results of our evaluation experiments on the testbed and compare them with simulated results when appropriate.

#### 5.2.1. Congestion Control Optimizations

Congestion control is an important aspect of group communication, especially in LLN, where resources are limited and network congestion can lead to extended response times and significant energy consumption due to frequent retransmissions of packets. CoAP provides basic congestion control by using the exponential back-off mechanism (Section 2.2.1) and by limiting the number of open requests from a client to any server to one request by default. Furthermore, CoAP specifies that, when using multicasts, a certain random delay should be inserted before replying to multicast requests. In CoAP terms, this delay is called leisure. The server could either use a default value for leisure or compute a value for it. If the server has a group size estimate *G*, a target data transfer rate *R* and an estimated response size *S*, a rough lower bound for leisure can then be computed as:

(1) $$Leisure_{lowerbound}=\frac{S\times G}{R}$$

In our experiments, *G* was between five and 40, *S* equals approximately 80 bytes and the target rate *R* can be set to a conservative 8 kbit/s = 1 kB/s. The resulting lower bound for the leisure is then between 0.4 s and 3.2 s. However, since CoAP servers will often not be able to compute the leisure, we elected to use the default leisure value of 5 s, as recommended by [6], in all of our multicast experiments. For a more complete discussion of the leisure period and its estimation, we refer to Section 8.2 of [6].

CoAP does not specify a congestion control mechanism when a single client is communicating with many servers using unicasts, as is the case in our group communication solution. However, our experience shows that this can quickly lead to congestion. A simple solution for avoiding network congestion when using unicasts is to limit the rate at which requests are sent. This way, the group members will get the requests spread over a period of time, and thus, there replies will also be spread over a period of time in a similar way to leisure. In order to get the replies spread over a period Leisure, the EM should insert a delay between requests *D* that equals Leisure divided by G-1, e.g.,

(2) $$D_{lowerbound}=\frac{Leisure_{lowerbound}}{G-1}=\frac{S\times G}{R(G-1)}$$

**Figure 7:**
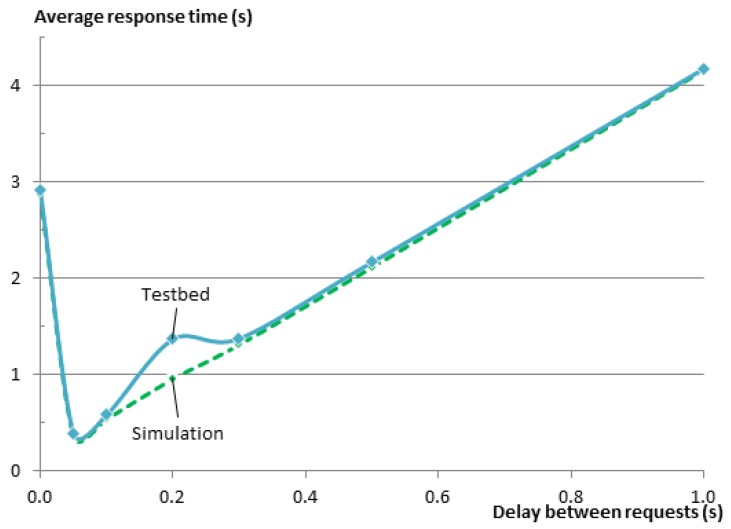
Response time of an entity with five members as a function of the delay between individual requests, evaluated using both a simulator and the experimental testbed.

For our experiments, we get $D_{lowerbound}=100$ ms and $D_{lowerbound}=82$ ms for G=5 and G=40, respectively. In order to verify the effect of the delay length, we conducted a series of experiments on the testbed to query an entity of five members and measure the response time, which is expressed as the time between the moment the client issues the request to the EM until it gets back the response. We repeated the same experiment for different delays between the requests sent from the EM to the members. We repeated the experiment 50 times for each setting and computed the averages. During these experiments, all Wi-Fi devices were turned off, and thus, no noticeable external interference was present. In [9], we have done the same experiments, but using the Cooja network simulator. Figure 7 shows the results of the experiments on the testbed and in Cooja. In general, there is a very good match between the results of the simulation and the results on the testbed. The figure clearly shows the need for the delay between the requests. Without inserting the delay, the response time of the entity was about 3 s. When using a delay of 0.1 s as calculated from Equation (2), the response time drops to 550 ms and is very close to the minimum value of 390 ms that was achieved for a delay of 50 ms.

In order to verify whether the same relationship exists between the delay and the response time for other group sizes, we have repeated the same set of experiments on the testbed using additional group sizes (g=10,20,30,40). At the time of experimenting with 30 members, one testbed node did not start properly, leaving only 29 group members in the experiment.

**Figure 8:**
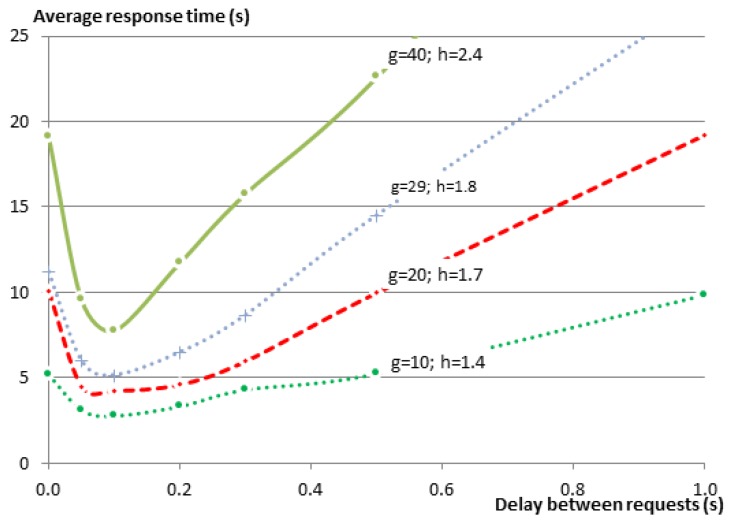
Entity response time for different group sizes as a function of the delay between individual requests, evaluated using the testbed.

**Figure 9:**
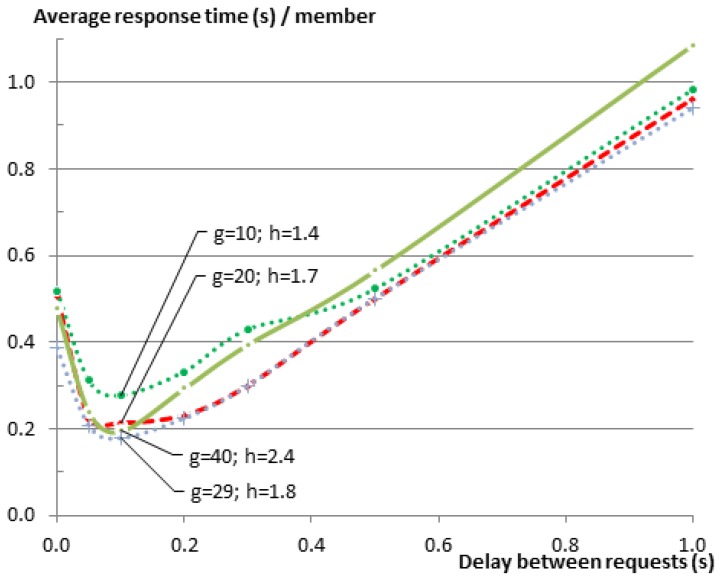
Entity response time per member for different group sizes as a function of the delay between individual requests to members, evaluated using the testbed.

Figure 8 shows the results of those experiments. Since the EM sends the requests to the members sequentially, it is expected that the response time for the complete entity gets larger as the group size gets larger. This relationship is very obvious in the figure. Regardless of this fact, one can see that graphs for all of the group sizes follow the same pattern. To further analyze the relationship between the delay and the group size, please consider Figure 9, which shows the same results as Figure 8, but this time normalized over the group size *G*. In a star topology, such as in our case, where all members need to communicate with the root of the star (the GW), one expects that the average response time would increase as the number of neighbors increases, resulting in a higher number of collisions on the shared medium. This is indeed the case when we compare any larger group size with the group of five members. However when comparing the larger groups together, this relationship cannot be observed. The reason for this is that with the increase of the group size and the way nodes are distributed over the testbed, members become no longer directly reachable from the GW, and their traffic was routed via other members. Consequently, the average hop count (*h*) was also increasing from just one hop for the group of five members to 2.4 hops for the group of 40 members, while the maximum hop count increased from one to five (see Table 7). A higher hop count implies that a lower percentage of members can communicate directly with the GW. It also means that a lower percentage of nodes is in the collision domain of the GW. This makes it possible that more parallel communication can happen inside the Wireless Sensor Network (WSN) before they reach the collision domain of the GW where the bottleneck is located.

Regardless of the small changes in the values for the various groups, we observe that the shape of the relationship function is very similar among all of them. The response times were always improved significantly when the delay was around the recommended range of 82 ms to 100 ms. However, as the delay between requests grows larger, it becomes the dominating factor for the total response time with a linear relationship between the two.

Another indicator of the performance of any communication solution is its reliability. During the tests we conducted in this section, the reliability of the communication was always 100% for all group sizes lower than 40. This is not surprising, since we had no external interference, and the only cause for errors was internal collisions. For the group size of 40, the reliability of member replies was never 100%. It was always between 99.8% and 99.9%, regardless of the delay between requests. This is also not surprising as with the larger group size, the chance for collisions increases, and the CoAP retransmission mechanism starts to be sometimes insufficient. We will discuss reliability in more detail in the next subsection.

As a result of the observations we made in these experiments, we have used an entity delay between requests of 100 ms in all of the following experiments, which is also in line with the results of Equation (2).

#### 5.2.2. Reliability

Reliability is a key performance indicator. In this subsection, we experimentally evaluate the reliability of both unicast and multicast CoAP group communication in the presence of Wi-Fi interference. To generate this interference, we send UDP traffic from one Wi-Fi node to the other at a constant bandwidth by using the iperf tool. We have setup the Wi-Fi communication to use Wi-Fi Channel 13, which completely overlaps with IEEE 802.15.4 Channels 25 and 26 that we use inside the WSN. Since we are using CSMA as the Media Access Control (MAC), the sensor nodes will back off when Wi-Fi is sending. However this is not true for the other direction. Typically, Wi-Fi MAC will not detect that wireless sensors are sending and will not back off.

**Figure 10:**
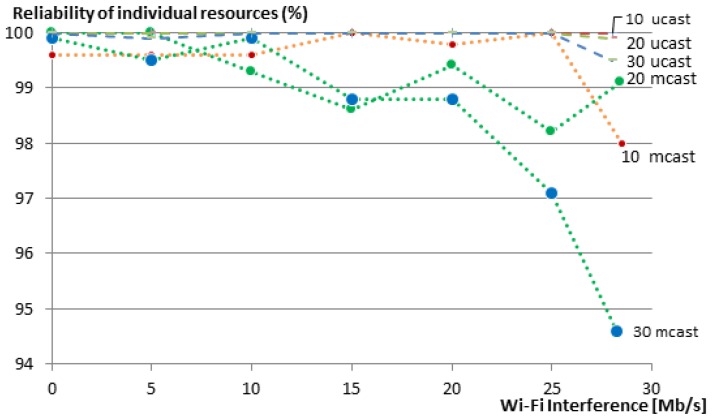
Member reliability for both unicast and multicast group communication and varying group sizes in the presence of Wi-Fi interference. The member reliability is much better when using unicast-based group communication.

To measure the reliability, we used the same experiment setup shown in Figure 6 to communicate with a group of 10, 20 and 30 members. We gradually increased the Wi-Fi interference in the network in steps of 5 Mb/s and measured the reliability of getting responses to the respective requests. We repeated the same experiment for our group communication solution and for multicasts. We run each experiment 50 times and show the averages of the member reliability (i.e., reliability of the communication with individual group members) in Figure 10. Multicasts are not transported reliably, and thus, the reliability of the network decreases as soon as there is a packet loss due to the Wi-Fi interference in the network. When using our unicast group communication solution, CoAP confirmable messages are used.

For the group of 10 members, the reliability of individual resources remains always 100%, even when the Wi-Fi nodes were transmitting as fast as they could (28.5 Mb/s). The reliability of individual resources for the group of 20 nodes dropped a bit to 99.9% under maximum Wi-Fi interference. For the 30-member group, the reliability is further reduced to 99.5% (compared to 94.6% in the case of multicasts). Figure 10 also shows that the reliability of individual members decreases with an increasing group size, both for unicast and multicast communication. This is due to two reasons. First, larger groups are denser and, thus, have a higher chance of collision between the group members. Second, and maybe with a higher impact on the reliability, bigger groups have a larger average hop count. This means that every message (both request and reply) between a client and a server has an additional chance of getting dropped at each hop on the way to its destination. Nevertheless, in our 20- and 30-member groups, 100% reliability was maintained for unicast communication until a Wi-Fi transmission rate of 25 Mb/s, with one single exception for the 30-member group at 5 Mb/s, where one message was lost and resulted in a reliability of 99.9%.

**Figure 11:**
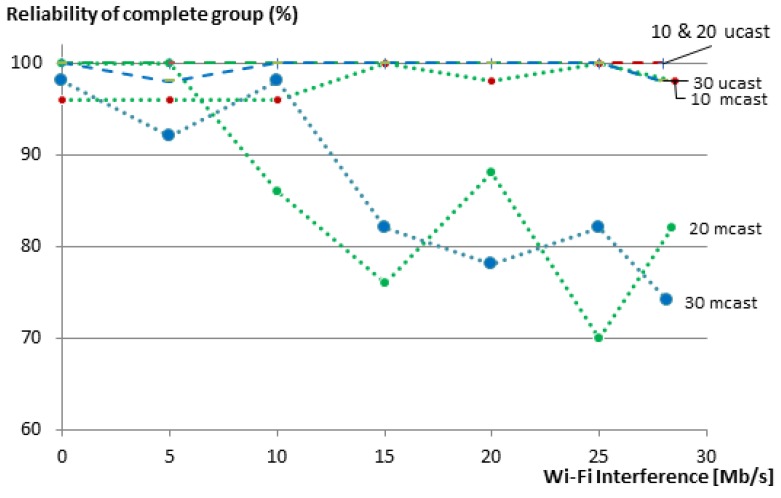
Entity reliability for both unicast and multicast group communication and varying group sizes in the presence of Wi-Fi interference. The reliability of the complete group is less than the reliability of individual members (Figure 10). Again, the reliability of the complete group is much better when using entity-based group communication.

In many group communication use cases, it is desirable to get answers from all members of the group. A complete group communication is considered successful when communication to all members in the group is successful. Figure 11 shows the effect of packet loss on the reliability of the complete group for our 10-, 20- and 30-member groups. Certainly, the reliability of a complete group is less than the reliability of its individual members, since the loss of a message to or from a single member renders the complete group request unsuccessful. In these cases, the use of multicasts does not provide good results. Already at 15 Mb/s Wi-Fi traffic, the reliability of 20- and 30-member groups drops to about 80%. In contrast, our unicast-based group communication maintains 100% reliability for the 10- and 20-member groups, even with the maximum transmission speed of the Wi-Fi nodes, and only drops to 98% in the case of the 30-member group.

These results are generally in line with the simulations that we performed previously in [9]. However, a direct comparison is not possible, since the simulations used a more controlled topology, in which five nodes were one-hop away, another five nodes were two hops away, and so on. On the testbed, the location of the nodes is fixed, and it was up to RPL to construct the topology for the routing. Furthermore, the simulations randomly dropped packets at a configurable percentage to simulate external interference, while on the testbed, real Wi-Fi traffic at one point of the network was used.

The drawbacks of the improved reliability of our unicast-based approach are the increased network overhead and response time. These are expected results, since the reliability is achieved by transmitting acknowledged messages, which results in more messages and longer delays in the case of errors. We have discussed these issues in detail in our previous work [9].

#### 5.2.3. Response Time

**Figure 12:**
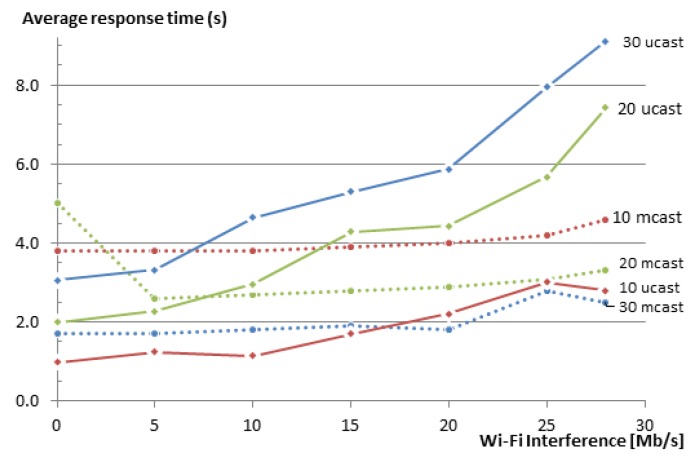
Entity response time for both unicast and multicast group communication and varying group sizes in the presence of Wi-Fi interference.

Another key indicator of the performance of any group communication solution is its response time. Figure 12 shows that the average response time when using our group communication solution increases with the increase of the Wi-Fi interference in the WSN. When there is no loss, the response time for unicast is just above the sum of the delays between the requests that the EM added, i.e., 1, 2 and 3 s for the groups of 10, 20 and 30 members, respectively. For multicast, the response time is determined by the leisure (5 s in our case). It is always lower than 5 s, since the nodes choose a value between 0 and 5 s before sending their replies. The value that is shown here is the value until the last response was received at the EM. When the interference increases, the response times for multicasts remain almost unchanged, since either the packet is delivered on time or it is just dropped. This way, the multicast solution is capable of maintaining lower response times at the expense of a decreased reliability. In the case of our solution, when a packet is dropped, CoAP attempts to retransmit it, leading to an increased overall response time.

These experimental results are also in line with the simulated results in [9]. Again, a direct comparison with the simulated results is not possible, as explained at the end of Section 5.2.2.

#### 5.2.4. Group Size

As shown in the previous subsections, using large groups can have a negative impact on the reliability of the group. In our tests, unicast groups started to become unreliable after a group size of 30 members. Multicast groups are generally unreliable, but the reliability also becomes worse with an increasing group size. The reason for this is that with the increase in group size, the density of the nodes typically also increases, and as a result, more collisions occur in the network. Furthermore, for the unicast-based solution, the group size directly affects the response time since the EM adds a delay between the requests it sends to the members. One simple solution is to split the groups. However, splitting the groups does not bring much benefit, when both groups are still using the same RF channel. When using our group communication solution, one can use more than one GW and create different WSNs that use different IEEE 802.15.4 RF channels. The groups are split accordingly.

**Figure 13:**
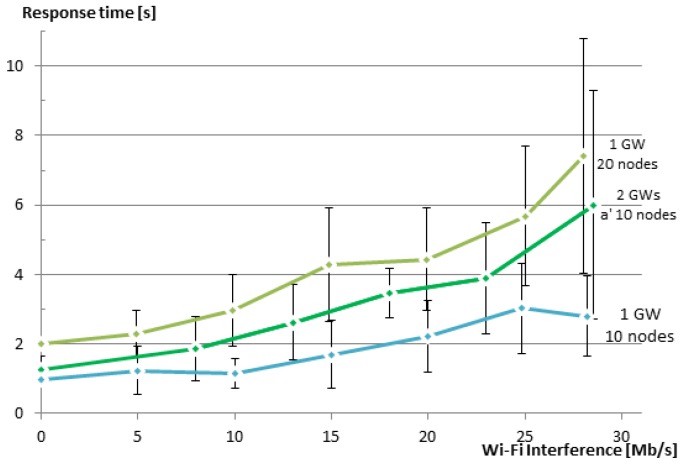
Entity response time in the presence of Wi-Fi interference for an entity of 20 members versus a nested entity consisting of two smaller entities having each 10 members.

In order to test this approach and to demonstrate the use of more than one GW to create two WSNs that are overlapping in the physical space, but are using different RF channels, we have created a new experiment. In this experiment, each GW is communicating with a network of 10 sensor nodes using its own RF channel (IEEE 802.15.4 Channels 25 and 26). The two GWs are connected via an Ethernet cable, and routing is enabled between them. We have repeated the same test as in Section 5.2.3, but now using a group that consists of two smaller groups. Figure 13 shows the response time vs. the speed of interfering Wi-Fi traffic for the new experiment along with the results for groups of 10 and 20 members from the previous section for comparison. As expected, the response time for the group of two smaller groups is better than that of the one big group, although the total number of nodes was the same in both cases (20 nodes). Further, the response time is larger than the case of a single group with 10 members. The reason here is that we use nested groups, i.e., a group that contains two groups. This results in some additional processing overhead and also inserts a delay of 100 ms between the requests being issued to the different subgroups. Additionally, since we used two neighboring IEEE 802.15.4 channels, a small amount of interference between the channels is present. The reason for selecting two neighboring channels was to have both channels equally interfered from the Wi-Fi Channel 13, which overlaps both of the used IEEE 802.15.4 Channels 25 and 26. In a production setting, one should not use a neighboring channel to also avoid this limited amount of interference. The selection of channels should also take into consideration which Wi-Fi channels are used.

#### 5.2.5. CoAP Retransmission Timeout

As described in Section 2.2.1, CoAP has its own basic reliability mechanism that can be used for unicast communication. When reliability is needed, the sender of the CoAP message should use a Confirmable Message (CON). The receiver has to acknowledge this type of messages by sending an ACK. If the sender does not receive a reply within a back-off time, it retransmits the confirmable message at exponentially increasing intervals, until it receives an ACK or runs out of attempts. By default, the initial back-off is set to a random time between ACK_TIMEOUT and ACK_TIMEOUT * ACK_RANDOM_FACTOR. By default, ACK_TIMEOUT = 2 s and ACK_RANDOM_FACTOR = 1.5, and thus, the default initial back-off is between 2 and 3 s. If a reply to the first transmission attempt of a CON is not received within the initial back-off time, the CoAP sender will double the initial back-off time and retransmit the packet. If a reply to the first retransmission is not received, CoAP will again double the back-off time and retry the transmission until MAX_RETRANSMIT (by default, four) is reached. If no reply is received after expiration of the back-off time of the last retransmission, the client will be notified about the error condition. When using the default values, the best case timeout will be after 2+4+8+16+32=62 s and in the worst case after 3+6+12+24+48=93 s.

**Figure 14:**
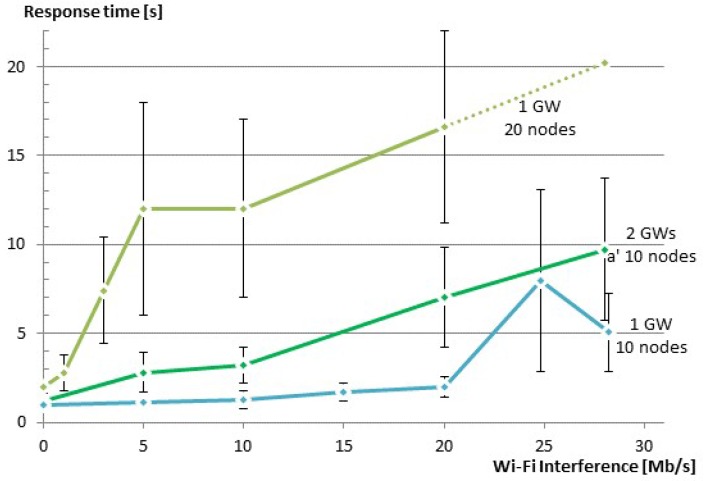
Entity response time of an entity with 10 members in the presence of Wi-Fi interference for varying initial back-off times.

The CoAP protocol allows the client to change the default parameters according to its needs. Changing those parameters will effect both the reliability and the response time. Changing MAX_RETRANSMIT effects the reliability directly, since it changes the number of attempts to get a successful communication. In our tests, the reliability was most of the times 100% with the exception of using large groups and large interference. As such, the default value of four retransmissions is fine for our use case. On the other hand, changing ACK_TIMEOUT, and thus, the initial back-off time, has a direct impact on the response time, since it specifies the time between the retransmission attempts. In order to investigate the effect of changing the initial back-off time on our solution, we have conducted a series of tests that are similar to those described in Section 5.2.2 for different values of the initial back-off times. Figure 14 shows the effect of Wi-Fi interference on the response time for three different values of the initial back-off time (ACK_TIMEOUT = 0.5, 1 and 2 s) for a group of 10 members. When there is no Wi-Fi interference, there is no need for retransmissions, and thus, the initial back-off has no effect. When Wi-Fi traffic was interfering with our WSN, reducing ACK TIMEOUT from 2 s to 1 s helped to improve the response time. However, reducing ACK TIMEOUT further to 0.5 s had a negative effect. This is due to the fact that in this case, CoAP was not waiting long enough for the replies to arrive and meanwhile trying to retransmit the requests, causing more collisions in the network.

### 5.3. Evaluation in a Real-Life Setup

The evaluations we have conducted so far were performed in a more or less controlled environment in an open-room testbed. This allowed us to analyze the behavior of the different group communication approaches in a clean environment with very limited external interferences. It also allowed us to inject controlled Wi-Fi interfering traffic and to study its effect of the WSN and the used group communication approaches. By doing so, we were able to compare the obtained results with the simulation results, which were also conducted in a comparable controlled environment. In some use cases, the radio environment might be clean, such as is the case inside a shielded room, such as our testbed. However, in many use cases, and in our building automation use case in particular, it is expected that much interference will be present that will affect the WSN. Typical WSNs operate in the Industrial, Scientific and Medical (ISM) radio bands. These radio bands are used by many other devices, not just WSNs and Wi-Fi, e.g., microwave ovens, cordless phones and Bluetooth.

**Figure 15:**
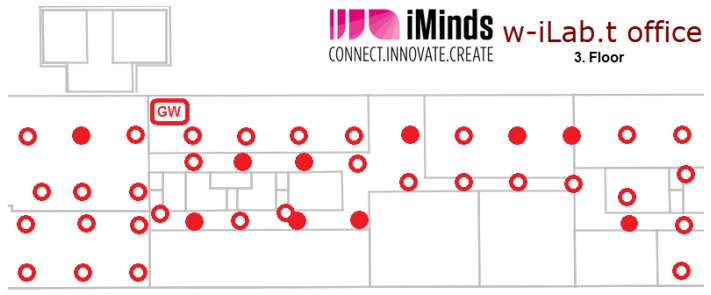
Experimental setup at the w-iLab.t office. The circles represent the location of the nodes on the third floor of the office building. The filled circles represent the nodes used in the experiment. The other nodes were idle.

In order to evaluate the behavior of our solution and that of the standard multicast solution in an environment that is more realistic than the open-room testbed, we have conducted a series of tests on the w-iLab.t office testbed. This wireless sensor testbed contains about 200 nodes spread over three floors (15 by 90 m each) of an operational office building in Ghent, Belgium [33]. In our experiments, we used 10 nodes spread over parts of the third floor during office hours (Figure 15). The circles represent the location of the nodes on the third floor of the office building. The filled circles represent the nodes used in the experiment. The other nodes were idle. As can be seen in the figure, the nodes are located in different rooms and in the hallways. In this setup, we have no control over the amount of interference that occurs during the experiments, since the sources of interference are diverse and are beyond our control; as is typically the case when using the Industrial, Scientific and Medical (ISM) radio band. A main source of interference in this part of the building is Wi-Fi, which was operating on Wi-Fi Channel 13. As explained before, this channel completely overlaps IEEE 802.15.4 Channel 26, which we used inside the WSN. Other sources of interference are also present (cordless phones, Bluetooth headphones and a microwave oven), but those are not always used.

We conducted two sets of experiments at two different times of the day in order to get different conditions for the tests. The first set of experiments was conducted when a few people were present at the office, and the other set was conducted when almost everybody was present and working. In each set of experiments, we queried the group of ten nodes 50 times using our unicast-based group communication solution and another 50 times using multicast. Similar to the experiments in the open-room testbed, we have measured the reliability of the communication and the response time. In Table 8, we summarize the results of these experiments. These results confirm the results we obtained from the experiments on the open-room testbed. Under normal network environments, multicasts reliability is not very encouraging. Replies were received from the members with a success rate of 91.2%, resulting in a poor 58% reliability for the complete group. On the other hand, the reliability of our solution was maintained at 100% since the CoAP retransmission mechanism was able to handle all errors. Of course, this leads to higher response times for the unicast groups, which increased from 1.7 s to 7.21 s on average between the two experiments. As expected, the response time for multicasts was not much affected.

**Figure 16:**
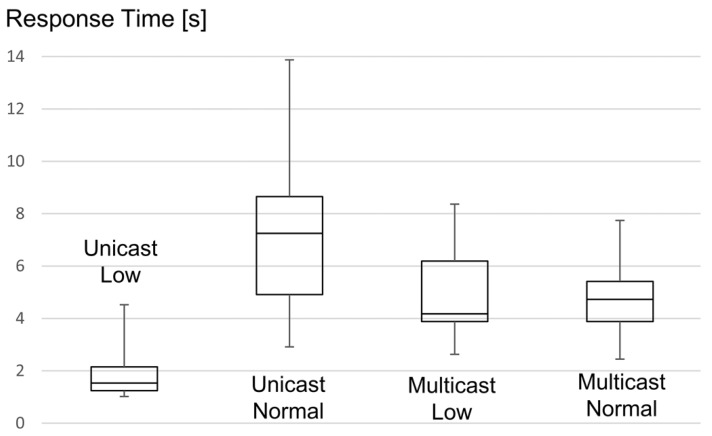
Entity response times for both unicast and multicast group communication in a real-life environment under low and normal network usage. Unicast reliability is achieved by exponential retransmissions and leads to a large increase in the response times.

In order to take a deeper look at the group response times, we summarize them in Figure 16 as Tukey boxes with whiskers and a central point, which are five-point summaries of datasets, corresponding to the minimum, 25th percentile, median, 75th percentile and maximum. Because of the well-defined back-off mechanism used by CoAP, it is possible to extract information about the amount of retransmissions, and thus, the reliability, from a Tukey boxplot of the latency. Looking at the box for unicast under low network usage, one can say that roughly in 70% of the cases, all group members replied after the first request, since the EM replied in less than 2 s and the first retransmission occurs after 2 s to 3 s. For the remaining 30%, at least one member of the group needed one retransmission. A second retransmission, taking place between 6 s and 9 s, was never needed. In the case of multicast communication, the latency is significantly higher because of the way we configured the leisure period. Here, every packet loss will directly result in a decrease of the group reliability. Under low network usage, the reliability dropped to 62%, which is more or less in line with the packet losses observed during the unicast experiments, which was executed under similar conditions. Now, looking at the box for unicast under normal network usage, we see that less than 25% of the cases required no retransmission. For the remaining 75% of the cases, one or two retransmissions were needed for at least one member of the group. A third retransmission, which would occur between 14 s and 21 s, was never needed.

### 5.4. Summary of Results

In this section, we have demonstrated that it is possible to implement all three suggested approaches for group communication (i.e., unicast, multicast and hybrid) on constrained devices. Our experimental results on two testbeds confirm our previous results that were obtained by simulations. These results show that it is essential to have mechanisms in place to avoid congestion in the LLNs. The mechanisms suggested by CoAP are a good starting point, but leave plenty of room for improvements. In general, the multicast-based group communication approach has less network overhead and low reasonable latency, but lacks reliability and security. On the other hand, our unicast-based solution is flexible, reliable and can be easily secured using standard CoAP methods at the cost of increased network overhead and latency. We have shown that our hybrid solution can be used to build customized groups that benefit from the pros of each approach to achieve a better overall group communication solution.

## 6. Conclusions and Outlook

The ability to communicate with groups of resources is important for many IoT applications in general and for BASs in particular. CoAP, which is expected to play an important role as an application protocol for use in constrained environment, does not have built-in group communication features. However, CoAP is designed in way that makes it easy to extend. Currently, there are two main approaches to extend CoAP with group communication capabilities. The fundamental difference between the two approaches lies in the underlying communication type: multicast versus unicast. The trade-off between the two communication types is reliability versus speed. In this paper, we proposed a hybrid solution that tries to get the benefits of both approaches. The solution is flexible to allow the user to select the communication type based on the desired features. As such, we believe that our solution is a powerful enabler for group communication in LLNs and an interesting building block for IoT applications.

Next to this, we have experimentally evaluated those approaches using two WSN testbeds (one testbed in a shielded room and another in an office environment). We explored several aspects (overhead, timing, scalability, etc.) related to the usage in realistic sensor networks and compared the different approaches. Experimental evaluation reveals that there are limitations to the size of the groups. This may impact the design of real BASs that typically operate in conditions where interference is inevitable. A further outcome of the experimental evaluation is the impact of the CoAP parameter settings (leisure, back-off, etc.) on the group communication performance.

During the evaluations, we have identified a number of possible paths for further optimizations and improvements. First of all, more in-depth research on different back-off strategies and congestion control mechanisms is needed in order to achieve an optimal balance between reliability and latency. Further, the possibility to nest entities allows one to construct entities that reflect hierarchies of IoT objects. Nested entities require further assessment in terms of performance. Different strategies for the breaking up of the entities should be explored. Finally, we believe that tighter integration with lower layers may further improve latency and reliability. Depending on the configuration of the groups and the type of resources involved, application requirements can be derived and translated into the optimal configuration of the lower layers, e.g., optimized MAC schedule, Time Division Multiple Access (TDMA), IPv6 over the TSCH mode of IEEE 802.15.4e (6TiSCH), local retransmissions, etc. This way, powerful IoT application enablers at the higher layers can be combined with advancements at the lower layers, together delivering the performance expected by IoT applications and their users.

## Figures and Tables

**Table 1:** Potentially relevant types of resource grouping in Building Automation Systems (BASs).

Type	Groups Based on	Example
Physical	physical structure of building	desk, part of room, room, wing, floor, building, etc.
Logical	similarity or functionality	all light intensity sensors
Application	application point of view	power consumption of heterogeneous devices
Administrative	roles, rights or policies	access keys of security guards

**Table 2:** Group communication requirements.

Requirement	Description
Flexibility	accommodate the differences between devices and device types
Light-Weight	limited footprint and should scale with the number of groups
Use of Standards	allows the creation of groups across members from different vendors
Performance	little overhead, efficient in the use of resources
Error Handling	mechanisms for reporting and handling error conditions
Reliability	sometimes it is not essential to get reliable replies from all group members
Ease of Group Manipulation	easily adapt groups based on changes of user needs
Expressiveness/Control	processing individual member results and replying with aggregated results
Security	compromising the group means compromising all of the individual members

**Table 3:** When multicast is enabled on Contiki nodes, they automatically join four link-local groups. RPL, IPv6 Routing Protocol for Low-Power and Lossy Networks.

Group IPv6 Address	Description
ff02::1	All nodes on the local network segment
ff02::2	All routers on the local network segment
ff02::1a	All RPL nodes on the local network segment
ff02::1:ff00:xx	Solicited-node multicast address. xx: node ID

**Table 4:** The group membership management resource /mc interface.

Method	URI Query	Request Payload	Description
GET	–	–	Show membership
POST	act = add	IPv6 address (array of bytes)	Join group
POST	act = del	IPv6 address (array of bytes)	Leave group

**Table 5:** Resource availability and requirements. The available resources on sample devices and the requirements for various system components. TM, Trickle Multicast; SMRF, Stateless Multicast RPL Forwarding.

	ROM (Bytes)	RAM (Bytes)
Class 1 device	≈100k	≈10k
Zolertia Z1	92k	8k
Rmoni RM090	256k	16k
TM (with one user defined multicast address)	4176	1802
SMRF (with one user defined multicast address)	1516	322
Additional multicast address	–	18
Multicast group management resource	1408	28

**Table 6:** Evaluation experiments settings.

Node Type	Rmoni RM090	
Contiki version	GitHub master branch: snapshot 2 July 2014	
Radio Duty Cycling (RDC)	Null-RDC	
Media Access Control (MAC)	Carrier Sense Multiple Access (CSMA)	
Routing	Protocol:	RPL
	Mode:	Storing
	Max neighbors:	50
	Max routes:	60
Multicast	Engine:	SMRF
	Max multicast addresses:	6
Radio Frequency (RF)	GW1:	IEEE 802.15.4 channel 26
	GW2:	IEEE 802.15.4 channel 25
	Wi-Fi 1 and 2:	Wi-Fi Channel 13
CoAP	Version:	Draft-18
	Leisure:	5 s

**Table 7:** Characteristics of the WSNs used in congestion control experiments on w-iLab.t Zwijnaarde wireless sensor testbed.

Number of Nodes	Hop Count (*h*)	
	Average	Maximum
5	1	1
10	1.4	2
20	1.7	3
29	1.8	3
40	2.4	5

**Table 8:** Summary of the results of the experiments on the w-iLab.t office wireless sensor testbed.

Communication Type	Network Usage	Reliability		Response Time (s)		
		Member	Group	Min.	Average	Max.
Unicast	Low	100%	100%	1.02	1.70	4.53
Unicast	Normal	100%	100%	2.91	7.21	13.87
Multicast	Low	92.2%	64%	2.62	4.92	8.36
Multicast	Normal	91.2%	58%	2.45	4.79	7.75
